# Prevalence of *Coxiella burnetii* and *Brucella* spp. in tissues from subsistence harvested northern fur seals (*Callorhinus ursinus*) of St. Paul Island, Alaska

**DOI:** 10.1186/s13028-014-0067-x

**Published:** 2014-10-01

**Authors:** Colleen Duncan, Bobette Dickerson, Kristy Pabilonia, Amy Miller, Tom Gelatt

**Affiliations:** Department of Microbiology, Immunology and Pathology, Colorado State University, Diagnostic Medical Center, 300 West Drake Avenue, Fort Collins, Colorado 80524 USA; National Marine Fisheries Service, Alaska Fisheries Science Center, National Marine Mammal Lab, 7600 Sand Point Way NE, Seattle, Washington 98115 USA; CytoVet, 5916 Willow Lane, Dallas, Texas 75239 USA

**Keywords:** Alaska, *Brucella*, *Callorhinus ursinus*, *Coxiella burnetii*, northern fur seal, zoonoses

## Abstract

**Background:**

The northern fur seal (*Callorhinus ursinus*) is an important cultural and nutritional resource for the Aleut community on St. Paul Island Alaska. In recent years, an increasing number of zoonotic pathogens have been identified in the population, but the public health significance of these findings is unknown. To determine the prevalence of *Coxiella burnetii* and *Brucella* spp. in northern fur seal tissues, eight tissue types from 50 subsistence-harvested fur seals were tested for bacterial DNA by real-time polymerase chain reaction.

**Findings:**

Of the 400 samples tested, only a single splenic sample was positive for *Brucella* spp. and the cycle threshold (ct) value was extremely high suggesting a low concentration of DNA within the tissue. *C. burnetii* DNA was not detected.

**Conclusions:**

Findings suggest that the risk of humans contracting brucellosis or Q fever from the consumption of harvested northern fur seals is low.

## Findings

On St. Paul Island Alaska, one of the Pribilof Islands, the northern fur seal (NFS, *Callorhinus ursinus*) is culturally significant to the Aleut community and an important traditional food. Historically infectious diseases have been considered an uncommon cause of mortality in this species [1], however since 2010 an increasing number of infectious agents have been identified [2-4], some of which are zoonotic.

In 2010 and 2011 *Coxiella burnetii* was identified in ~75% of the NFS placentas collected from a NFS rookery [3,5]. *C. burnetii* is a zoonotic bacterium that can cause a wide range of disease in humans including generalized malaise, severe multi-systemic disease and chronic valvular endocarditis [6]. Inhalation is thought to be the most common mode of transmission, but the organism can also be spread by tick vectors and ingestion [6]. Milk is the primary source of food-borne *C. burnetii*; an infection source that had decreased with pasteurization but has recently been increasing with the ‘raw milk’ movement [7]. While the risk of contracting disease following consumption, or handling, of other tissues is less understood, disease outbreaks have occurred in slaughterhouse and meat processing facilities [8].

*Brucella* has also recently been identified in NFS placentas and in serum from an individual animal that was harvested for human consumption [2]. The *Brucella* genus includes a number of pathogenic species that have the ability to cause disease in both humans and animals. *Brucella* spp. have been isolated from a number of marine mammal species (recently reviewed in [9]) and marine mammal isolates have been shown to be pathogenic in people [10-12]. Brucellosis has been described as one of the most important infectious diseases in Alaska [13].

In humans and animals, *Brucella* spp. can persist in cells of the mononuclear phagocyte system such as the bone marrow, lymph nodes, spleen and liver [14]. Similarly, chronic infection and long-term persistence of *C. burnetii* has been identified in the bone marrow and liver of humans many years after primary bacteremia [15,16]. It is presumed that a similar pathogenesis exists in marine mammals, but this has not been substantiated. The objective of this study was to determine the tissue distribution and prevalence of *C. burnetii* and *Brucella* spp. in tissues from NFS harvested for consumption on St. Paul Island, Alaska as these zoonotic pathogens may present a human health risk.

From July 5th to August 7th 2013 tissues were collected from 50 sub-adult male NFS during the St. Paul community harvest. Fresh samples of muscle, liver, kidney, spleen, testicle, lymph node, lung and bone marrow were collected and frozen at -80°C in 1.5 ml cryovials until testing. Bone marrow impression smears were made from the femur within 5 hours of the animals’ death and stored dry until transferred to the laboratory where they were stained with Wright-Giemsa Stain (Volu-Sol Inc., Salt Lake City Utah, USA). A fresh dog bone marrow sample was stained with the NFS samples as a positive staining control and samples were excluded when ≥ 75% of the cells were autolytic. A section of formalin fixed bone marrow was routinely processed and examined histologically.

The eight tissue types (n = 400 samples) were individually tested for both *C. burnetii* and *Brucella* spp. by real-time polymerase chain reaction (PCR). DNA was extracted from all tissues using the QIAamp DNA Mini Kit (Qiagen, Valencia, California, USA) according to manufacturer’s directions with the following exceptions: 80-120 mg of tissue was used as the starting sample and the sample was incubated at 56°C in proteinase K overnight for 16-18 hours. Real-time PCR was conducted as previously described for the *C. burnetii* IS1111 and COM1 gene target regions [3] and *Brucella* spp. IS711 gene target region [2]. A sample was considered positive at a cycle threshold (ct) value of less than or equal to 40. Marine mammal origin *C. burnetii* and *B. pinnipedialis* positive amplification controls, and a no template negative control, were used for the *Coxiella* and *Brucella* real-time PCR respectively.

A single splenic sample was positive for *Brucella* (ct value 38.52); this result was repeatable but all other tissue types from this individual were negative. Of the remaining 399 samples, all were negative for both bacteria. Results of the bone marrow cytology are presented in Table 1. A single animal had a significantly higher myeloid: erythroid (M:E) ratio suggestive of granulocytic hyperplasia but this animal was PCR negative on all tissues. No histologic lesions were identified in the bone marrow of any animals.

**Table 1:** **Nucleated cell differential counts from 20 sub-adult male NFS bone marrow samples examined cytologically**

	M:E ratio*	**Lymphocytes (%)**	**Plasma cells (%)**	**Macrophages (%)**	**Megakaryocytes**
**Mean**	2.14	1	0	1	6.6
**Median**	1.45	0	0	1	6.6
**Range**	0.8-7.0	0-4	0-2	0-4	1.8-12.2

*The M:E ratio and% lymphocytes, plasma cells and macrophages were based on a 200 nucleated cell count in cellular areas of the slide with minimal artifact. The megakaryocyte count was based on the average of 5 cellular 10x fields.

Results of this study suggest that the risk of exposure to *C. burnetii* and *Brucella* spp. through subsistence harvesting of male NFS on St. Paul Island is extremely low; only 0.25% of tested samples (one animal) had evidence of bacterial DNA in tested tissues. The single *Brucella* positive spleen sample had a high ct value suggesting a low quantity of DNA within the tissue; follow up attempts to culture *Brucella* from the splenic tissue of this animal were unsuccessful. Given that no other tissues from this animal were positive it is doubtful that this animal had an active infection, although the positive result could represent inactive DNA persisting within a splenic macrophage. As the spleen is not routinely collected or consumed by the Aleut people, this finding is of minimal public health concern. The apparent *Brucella* prevalence of 2% in this study is consistent with the previously reported placental prevalence (5%) and seroprevalence (2.5%) in this species [2]. While brucellosis has been identified as an important zoonotic disease in Alaska, the public health risk is thought to be from the hunting of infected terrestrial, and not marine, mammals [13].

That all samples were negative for *C. burnetii* was unexpected. The 2011 *C. burnetii* seroprevalence in subadult males was 73% [17] suggesting that exposure is high in the cohort of animals included in the present study. Failure to identify any tissues positive by PCR suggests that for exposed NFS the infection rate is low, infection is transient or the bacteria persists in tissues other than those tested in this study. The negative findings were also surprising given recent reports of human exposure in the area. Archived serum from residents of St. Paul Island had *C. burnetii* antibodies in 12% of the samples (titer ≥ 1:64) [18], which is significantly higher than the general US population (3% at titer ≥ 1:16) [19]. The present study suggests that this differential exposure is unlikely to be from consumption of NFS, however discordance between sampling timeframes that makes it challenging to compare these results. The archived human serum tested was collected between 1980-2000 and individuals included in that study may have had more opportunities for *C. burnetii* exposure. The commercial NFS harvest was active on St. Paul Island until 1985, with tens of thousands of animals killed annually for furs providing considerably more human-animal interaction [20]. Following the ban on commercial hunting, the Aleut people continued to hunt NFS for subsistence use although the average annual harvest has been less than 500 sub adult males over the last 15 years. Another possibility for the discordant results is that human exposure is coming from inhalation of aerosolized organisms arising from the contaminated birthing rookeries of the NFS. While it is illegal to disrupt federally protected rookeries during June to Mid Oct while NFS are on land, the organism can persist year round in these locations [5] allowing for exposure opportunities even during periods when no animals are on the island.

Bone marrow was examined both cytologically and histologically in this study because chronic *C. burnetii* infection in humans has been associated with bone marrow pathology such as ‘doughnut ring’ granulomas [21,22] and an acute hemophagocytic syndrome [23]. While neither of these lesions were observed, results of the bone marrow analysis may be helpful as a reference range for this species given the paucity of baseline information on many free ranging pinnipeds. The results of the cytologic examination of the bone marrow were similar to the reference ranges for other common domestic species. In addition, the good preservation of many of the bone marrow samples collected up to 5 hours postmortem suggests that significant autolysis may not occur as quickly in this species as compared to others.

While the present study suggests that there is little risk of human exposure to *C. burnetii* and *Brucella* spp. through the subsistence harvest of NFS on St. Paul Island, there remain some unanswered questions regarding the source of human exposure to *C. burnetii* on the Pribilof Islands of Alaska. Given that little is known about this zoonotic agent in the marine and coastal environment, further investigation into the epidemiology and ecology of this pathogen will help to guide public health recommendations.
